# IV3TM: Inception V3 enabled bidirectional long short-term memory network for brain tumor classification

**DOI:** 10.1371/journal.pone.0335397

**Published:** 2025-10-24

**Authors:** Afnan M. Alhassan, Nouf I. Altmami

**Affiliations:** Department of Computer Science, College of Computing and Information Technology, Shaqra University, Shaqra, Saudi Arabia; Dayananda Sagar University, INDIA

## Abstract

A brain tumor is one of the life-threatening neurological conditions affecting millions of people worldwide. Early diagnosis and classification of brain tumor types facilitate prompt treatment, thereby increasing the patient’s chances of survival. The advent of Deep Learning methods has significantly improved the field of medical image classification and aids neurologists in brain tumor diagnosis. However, the existing methods using Magnetic Resonance Imaging (MRI) face significant difficulties due to the complexities of brain tumors and the variability in tumor characteristics. Consequently, this research proposes the Inception V3 enabled Bidirectional Long Short Term Memory Network (IV3TM) for Brain Tumor Classification. In the proposed approach, the preprocessing and data augmentation techniques are presented to enhance classification performance. At the pre-processing stage, an iterative weighted-mean Filter approach is utilized to cope with bias field-effect fluctuations, noise, and blurring in input images to enhance the edges. Further, the data augmentation strategy increases the size of the available training data. SqueezeNet is used to segment images for further classification operations. Further, the proposed model combines the strengths of Inception V3 and BiLSTM to learn the sequential dependencies significant for understanding the intricate structural relationships in brain MRI data. The effectiveness of the proposed method is evaluated using several metrics, including specificity, accuracy, precision, F1-score, and sensitivity. Furthermore, the proposed method’s error is evaluated using root mean square error (RMSE). Experiments using the Brain Magnetic Resonance Imaging (MRI) images dataset and Figshare brain tumor datasets have shown encouraging results.

## 1. Introduction

The brain is the human body’s most important and structurally complicated organ, which is covered by the skull layer, making it challenging to observe the behavior and illness diagnosis [1,2]. The condition of the brain differs from any other part of the body, although it may be brought on by abnormal cell development, which eventually damages the brain’s structure and results in brain cancer [3]. One of the most hazardous illnesses is a brain tumor, which may strike anybody, regardless of age, gender, or race. A brain tumor is an abnormal growth of brain tissue that develops from tissues that are connected to the brain [4], classified into benign and malignant. Benign tumors are non-cancerous, and Malignant tumors are cancerous, rapidly expanding, and fatal, which may conflict with regular brain activity [5–7]. The sensitive regions of cancer cells are the primary and secondary tumor sites. Primary tumors are curable, and their growth can be slowed down by taking the appropriate treatments [8]. Brain tumors that have progressed to the brain from another region are referred to as secondary (metastatic) brain tumors [9,10]. If the patient desires the necessary surgery or treatment, this tumor will be curable. Since brain tumors damage the surrounding brain tissue, it is important to continuously monitor their development to guarantee the patient’s life. Brain tumors are divided into four types by the World Health Organization: type I, type II, type III, and type IV [11]. Among all types, the type I tumor spreads slowly. However, the most deadly and destructive type of malignancy is a grade IV tumor. Thus, to improve patient survival, early diagnosis of tumors in the brain is important. The expertise of neurologists is required for accurate tumor identification [12,13].

Recently, computerized brain tumor diagnosis has become necessary due to the rapidly rising number of occurrences of brain tumors. For diagnosis, MRI is a commonly used tool for tumor analysis with its high-quality brain images [14]. The MRI is particularly important because it offers a distinctive manner to the very best-suited visualization of maximum contrast and spatial resolution [15–17]. Moreover, calculating the extent of a tumor and determining the level of ambiguity in the segment region are challenging due to anatomical complexity, unpredictability, and high volatility of brain tumors [18,19]. The technique of segmenting the tumor region into separate sections is known as image segmentation. In brain tumor detection, segmentation identifies the appearance or disappearance of a tumor in the image, which is labeled [20,21]. Brain tumor detection methods are divided into Machine learning (ML) and deep learning (DL) methodologies. A machine learning model is utilized to complete a task without using specific instructions, but rather by relying on multiple algorithms. As a result, the identifying accuracy of methods is dependent on the quality and representation; it performs well with limited resources and is restricted when coping with large datasets [22,23]. Meanwhile, DL-based models have outperformed others in a range of disciplines, which is a subset of ML that relies on understanding intricate information and learning hierarchical features [24]. DL incorporated versatile and very efficient approaches to solve medical image analysis tasks. Automatic segmentation on a large number of MRI datasets will be done effectively using DL approaches [25,26]. This research introduced a computerized technique for brain tumor segmentation and classification by utilizing MRI and incorporates advanced DL models. The hybrid technique incorporated the benefits of the most effective and widely used image classification techniques.

Nevertheless, the existing models utilized for brain tumor detection struggle with limited resources, overfitting issues, high computational complexity, poor generalizability, and vanishing gradient issues. The research gap addressed in this work lies in the persistent challenge of poor performance in existing deep learning models for brain tumor detection using MRI images. Many current methods rely on single-architecture models that either cannot effectively segment tumor regions or fail to capture complex spatial and contextual features necessary for accurate classification. This study introduces a novel hybrid approach combining SqueezeNet for efficient segmentation with an Inception V3–BiLSTM framework for robust feature extraction and classification. Unlike conventional models, this architecture distinctly separates segmentation and classification tasks, allowing each to be optimized independently. It improves upon existing methods by enhancing edge preservation during preprocessing, expanding training diversity through data augmentation, and leveraging BiLSTM’s sequential modeling capabilities to better capture inter-region dependencies. As a result, the proposed approach significantly boosts classification accuracy while reducing computational complexity and model overfitting, offering a more reliable and scalable solution for brain tumor diagnosis with better generalizability.

### 1.1. Problem statement

Medical practitioners can choose the appropriate treatment plan, which involves surgical treatment, chemotherapy, radiation treatment, or a mixture of these therapies, with the aid of the classification of tumors. The categorization of brain tumors is confounded by the presence of fluctuating tumor appearance and location, as well as noisy and complex imaging data. All medical image processing techniques still rely on segmenting the images, which involves removing regions of interest from the images. Given the enormous quantities of data that each photograph gives, this process takes too long, is tiresome, and even difficult.

### 1.2. Contribution of the work

This article provides a classification of brain tumors utilizing the incorporation of InceptionV3 with DBLSTM for identifying various tumor abnormalities to overcome the concerns highlighted above. The pre-processing stage of the initially proposed approach helps reduce bias field effect, noise, and blurring changes in images and enhances the edges in MRI. Second, a segmentation technique is used to isolate the site of the brain tumor from its surroundings. The InceptionV3 model is then used to extract the significant features from the segmented images. Last but not least, the deep learning DBLSTM model is recommended for the choice of important characteristics and tumor type classification.

The research’s key contributions are explained as follows:

***Inception V3 enabled Bidirectional Long Short Term Memory Network (IV3TM):*** In the proposed approach, the IV3TM model combines the strengths of the Inception V3 and DBLSTM to improve the hierarchical feature representation and effectively captures the sequential information and long-range dependencies within input MRI data to improve the classification accuracy. As a result, the proposed model effectively processes the spatial and contextual features for understanding the complex structural relationships in brain MRI data and contributes to improved brain tumor classification.

The remaining sections in the manuscript are listed below. Section 2 examined the existing methods for classifying brain tumors. Section 3 describes the entire framework of the proposed model. The fourth section discusses and analyses the experimental outcomes. Section 5 ends with conclusions.

### 1.3. Research motivation

The motivation behind using separate models for classification and segmentation in the proposed hybrid approach lies in optimizing performance by leveraging the strengths of specialized architectures for distinct tasks. Segmentation and classification involve fundamentally different operations—segmentation requires precise, pixel-level localization of tumor regions, while classification demands robust extraction and interpretation of high-level features. By employing SqueezeNet for segmentation, the model benefits from fast, parameter-efficient processing that accurately delineates tumor boundaries. In contrast, Inception V3 combined with BiLSTM is well-suited for capturing complex spatial and contextual features necessary for accurate classification. A unified model might compromise on one task to accommodate the other, potentially reducing overall effectiveness. The hybrid strategy enhances modularity, allowing independent tuning of each stage, and results in superior accuracy, computational efficiency, and adaptability to variations in brain MRI data, addressing the critical need for precision in medical diagnosis.

## 2. Literature survey

Many types of research have been published in recent years that show various strategies for segmenting, detecting, and classifying tumor areas utilizing MR brain imaging. Various frameworks are investigated for the identification of brain tumors; only a few of them are discussed in this section.

Sadad et al*.* [27] introduced a model for classifying and detecting brain tumors utilizing cutting-edge deep learning methods. The concepts of processing, as well as information augmentation, were added to improve the classification rate. On the Figshare data sets, segmentation utilizing Unet design with ResNet50 as its basis is demonstrated in this study. To extract important characteristics from MRI slices, two methods of Transfer learning (TL), “freeze” and “fine-tune,” are employed. NASNet architecture, ResNet50-UNet, and transfer learning are used for brain tumor multi-classification. Brain cancers are divided into several groups using evolutionary algorithms and transfer learning.

Khairandish et al. [28] recommended using a hybrid CNN- Support Vector Machine (SVM) approach to identify and categorize cancers in brain MRI scans. This research work has applied a hybrid technique using brain MRI images to recognize and categorize the tumor utilizing the BRATS database. The system in place uses to categorize brain images as either cancerous or normal tumors, trained mixed SVM and CNN methods were used. The next stage is pre-processing, which involves resizing the image and doing the filtration process. Without utilizing custom models, CNN may extract features using a variety of ways. After the feature has been retrieved, the image segmentation procedure is started. The model will undergo thorough feature extraction and segmentation before SVM and CNN training.

Toğaçar et al. [29] proposed the BrainMRNet CNN model. This structure, which relies on parts for attention and hypercolumn technology, employs a residual network. First, BrainMRNet does image preprocessing. Then, utilizing image augmentation methods for each image, this process is conveyed to attention modules. Convolutional layers receive the images after attention modules have chosen key portions of the image. One of the most crucial methods for using the proposed method within the convolutional layers is the hyper column. The final layer’s array structure inside the BrainMRNet model retains the characteristics that were taken from each layer using this method.

Saba et al*.* [30] presented the Grab cut approach, which is used to accurately separate the symptoms of a lesion. While the VGG-19 of the Transfer learning framework is updated to gain the handcrafted features, these characteristics are then integrated with these features utilizing an iterative technique. Using MRI, the glioma is segmented using the GrabCut approach. One of the most popular MICCAI challenge datasets is the multimodal brain tumor segmentation used to evaluate the provided approach. To classify the gliomas and healthy photos, Handcrafted characteristics like Histogram of Oriented Gradients (HOG), deep learning, and Local Binary Pattern (LBP) are extracted for improving the feature representation.

Ameer and Deepak [31] developed an automatic classification of tumors from MRI utilizing SVM and CNN features. Applications for image classification have been implemented using DL and ML models, and the results are encouraging. However, the small number of medical image databases constituted a limitation of medical image categorization. The researchers combined deep learning methods for medical data categorization to address this problem. The goal of CNN is to identify characteristics in brain MRI images. The model was enhanced using multi-classification. A four-fold cross-validation method was applied to test and assess the integrated system. To evaluate the model’s performance, extensive tests are run on various brain images*.*

Shakeel et al. [32] developed a brain tumor classification approach based on ML-based neural networks with backpropagation (MLBPNN), which allows pathologists to improve accuracy and competence in threat location while limiting the entomb onlooker diversity. Several image preparation procedures are needed to identify diseases from biopsy images. To reduce complexity, the characteristics of retrieved employing a dimension with fractals strategy, as well as the most significant features, are then chosen utilizing a multifractal detection method. The improved function aids in tumor pinpointing. The tumor’s area is computed, and it is then classified as a type I or type II tumor, with its accuracy also evaluated.

Sultan et al. [33] to identify several forms of brain cancers utilizing two publicly available datasets, a CNN-based DL model is given. In the first and second datasets, which consist of 516 and 3064 images overall on T1-weighted contrast-enhanced images, respectively, 233 and 73 patients from each group are included. Preprocessing is done on the images before feeding them into the CNN model. Further, the CNN model has 16 levels, beginning with an input layer storing the processed images and progressing through the convolution layers and their activation methods. Two layers of dropouts are also used to prevent overfitting. The output is then forecasted using a softmax layer and an entirely connected layer, and the predicted class is created using a classification layer.

Zulfiqar et al. [34] developed a DL architecture using two freely available resources or datasets using a CNN to distinguish different types of brain cancers. For effective preparation of the input photos in this study, they used a 2-D FIR filter based on the Tree Seed Algorithm. To classify photos, a Multi-Layer Perceptron (MLP) is employed, whereas the histogram is utilized to extract characteristics from the images. It is then divided using a tailored neural deep network structure into several classes.

Masood et al. [35], towards efficient Brain tumor classification and segmentation, customized Transfer learning was used to evaluate Mask RCNN with a denseness-41 backbone structure. Mask-RCNN, in conjunction with DenseNet-41, here tries to achieve effective localization, segmentation, and classification of tumors from MRI images despite the presence of bias field effect, blurring, and noise oscillations in the images. To extract the appropriate feature information from the input MR images, the backbone network is used. We were able to achieve better classification and segmentation results with the DenseNet-41 network owing to its dense relationships, which enable more precise image feature computations.

Al-Saffar et al. [36] recommended enhancing the results and lessening the difficulties associated with the analysis of medical photographs. To make an accurate diagnosis, brain tumors must be manually segmented, identified, and categorized based on MR images. For brain tumor segmentation, Local Difference in Intensity – Means (LDI-Means) is utilized, Mutual Interaction (MI) is utilized for selecting features, Singular Value Decomposition (SVD) is employed for dimensionality reduction, and SVM and MLP are employed for tumor classification. Furthermore, this study proposed a new strategy for selecting the most essential qualities as classifier inputs called Multiple Eigenvalues Selection (MES). The use of both unsupervised and supervised methods resulted in a good strategy for classifying brain gliomas.

Table 1 shows the overall summary of the existing methodologies from recent papers using MRI images. To tackle the various challenges raised in the previous research, our work focuses on the unique segmentation and classification approach proposed for the identification of brain tumors. Furthermore, the presented classification results show the greatest accuracy.

**Table 1:** Summarization of existing papers on brain tumor detection from MR images.

References	Technique	Limitations
Sadad et al. [27]	NASNet + Unet	Overfitting
Khairandish et al. [28]	hybrid CNN-SVM	High computational time
Toğaçar et al. [29]	BrainMRNet	Limited dataset & miss small tumors
Saba et al. [30]	VGG-19	Limited applicability
Deepak & Ameer [31]	CNN + SVM	Low data rate
Shakeel et al. [32]	MLBPNN	Challenging to optimize
Zulfiqar et al. [34]	CNN	Computationally intensive, requiring powerful hardware and significant time to train
Masood et al. [35]	Mask RCNN	Limited data
Al-Saffar et al. [36]	Multiple Eigenvalues Selection (MES)	Limited scalability and adaptability

## 3. Proposed inception V3 enabled bidirectional long short term memory network for brain tumor classification

**Fig 1:**
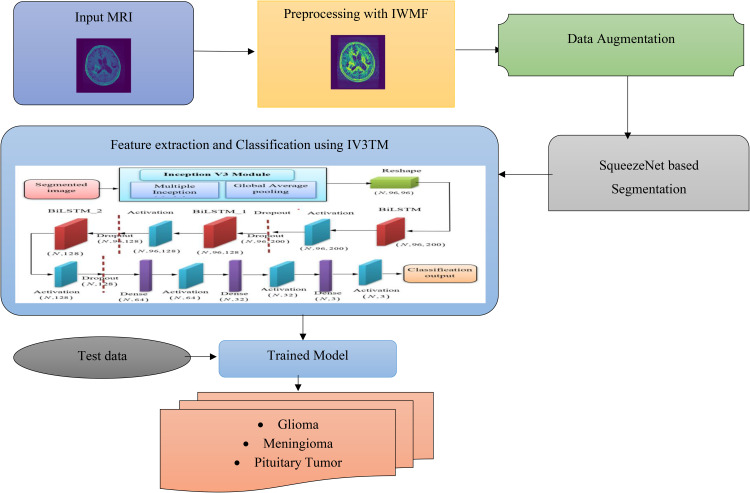
Workflow of the Proposed IV3TM model for brain tumor classification.

The proposed methodology consists of Pre-processing, segmentation, feature extraction, and classification. Initially, the input MR images are collected from the datasets and applied in the preprocessing stage. Here, an iterative weighted-mean filter approach (IWMF) is used to generate the improved image, and blur removal for image smoothening. Further, the preprocessed image is forwarded to a data augmentation technique to handle inadequate training data. In segmentation, the augmented image is segmented using the SqueezeNet, where the impacted tumor region is separated. After segmentation, the image is processed through feature extraction for learning significant features. Inception-V3 is used for feature extraction. Finally, DBLSTM is employed for reliable and precise classification. The brain tissues are classified here as Glioma, Meningioma, and Pituitary Tumors. While hybrid deep learning models combining convolutional and sequential architectures are increasingly common in the literature, the specific integration of Inception V3 with Deep Bidirectional Long Short-Term Memory (DBLSTM) in this work is tailored to address critical challenges in brain tumor classification. Inception V3 excels at multi-scale spatial feature extraction due to its parallel convolutional pathways, capturing fine-grained tumor patterns. DBLSTM, chosen over other sequential models like GRU or unidirectional LSTM, offers enhanced temporal dependency modeling by processing image-derived features in both forward and backward directions, effectively capturing contextual relationships between regions in segmented MRI slices. What sets this combination apart is its application to post-segmentation analysis, where DBLSTM leverages the sequential encoding of spatial features from the Inception backbone, enabling a more robust representation for classification. The manuscript can be strengthened by emphasizing how this bidirectional temporal modeling complements the spatial depth of Inception V3, thus enhancing diagnostic accuracy while mitigating vanishing gradient issues. Fig 1 illustrates the architecture of the proposed model utilized for brain tumor classification.

### 3.1. Input data

Initially, the input data is collected from the Figshare brain tumor dataset [37] and the Brain MRI dataset [38], which are utilized in the proposed IV3TM model for brain tumor classification. The paper uses publicly available datasets. The dataset description is explained as follows.

#### 3.1.1. Figshare brain tumor dataset.

The Figshare brain tumor database is an open data collection that is widely utilized for research purposes in medical image classification and retrieval [37]. We used Figshare’s public brain tumor data collection, which includes 3,064 brain MRI slices from 233 subjects. The Figshare dataset’s images are organized into five subdirectories. It comprises 3,064 axial, sagittal, and coronal MRI slices extracted from 233 patients, covering multiple tumor types and grades. Each image is formatted in high resolution (512 × 512 pixels) and stored in.mat files, which include not only the raw MRI data but also associated metadata like tumor type, coordinates of tumor boundaries, and binary ground truth masks for a precise segmentation task. The MRI images are in matrix form and have a 512 × 512-pixel resolution. The patient ID, as well as 512 × 512 image data in unsigned integer 16-bit format, brain tumor forms, tumor border with coordinate points, and binary mask in ground truth image, are all included in each MAT file. The dataset consists of 1426 MRI images in unbalanced and multi-view slices. Further, this dataset supports multi-modal imaging and contains different contrast-enhanced views, including T1, T2, and FLAIR sequences. The clinical diversity of patients, including variations in age, gender, and tumor pathology, enhances its robustness for developing generalized models. In this study, to maintain patient-wise independence between training and testing sets, a five-fold cross-validation strategy was employed, ensuring no patient data leakage across folds. Each fold retained an approximate class balance and allowed for comprehensive performance evaluation over unseen patient records. The richness in spatial detail, labeled segmentation masks, and clinical annotations make this dataset ideal for validating both classification and segmentation methods in real-world scenarios.

#### 3.1.2. Brain MRI dataset.

The brain MRI scans were taken from open-source datasets on Kaggle.com [38]. This study makes use of downloaded MRI-scanned image-based datasets. The proposed approach is fed the generated datasets. The approach to deep learning is primarily employed to find and classify brain tumors. As a result, massive datasets can be used to provide MRI-based brain cancer classification. Before augmentation, the total number of input images used was 248, while the total number of input images used after augmentation was 2073. The data is organized into two folders. The first folder contains 98 images that do not have tumors, while the second folder contains 155 images that do. The network is largely trained on MRI scans from the dataset. The functionality of the device is then assessed using photographs of normal and cancer tissue. The class imbalance in the Brain MRI dataset, with significantly fewer non-tumor images compared to tumor images (98 vs. 155), poses a critical challenge that can lead to biased model performance, particularly inflating metrics like accuracy while misclassifying minority class instances. To address this issue and ensure balanced learning, techniques such as oversampling of the minority class and class weighting during training were considered. Oversampling was performed using data augmentation strategies (e.g., rotation, flipping, scaling) specifically applied to non-tumor images to synthetically increase their representation. Additionally, class weights were incorporated into the loss function to penalize misclassifications of the underrepresented class more heavily. These measures collectively helped mitigate the skewed distribution, improving the model’s sensitivity and specificity across both tumor and non-tumor classes, thereby enhancing the reliability and fairness of the classification outcomes. The Brain MRI dataset obtained from Kaggle is a curated, publicly accessible dataset specifically designed for binary classification of brain tumors (tumor vs. non-tumor). It consists of 248 grayscale MRI images in JPEG/PNG format before augmentation, sourced from routine diagnostic scans. These images are uniformly resized to a standard input dimension (e.g., 240 × 240 or 299 × 299 pixels) to align with the input constraints of deep learning architectures. The dataset is notably class-imbalanced, with 98 non-tumor and 155 tumor images. To address this, data augmentation techniques such as affine transformations, Gaussian noise injection, histogram equalization, and elastic deformations were selectively applied to underrepresented non-tumor images, expanding the dataset to 2073 images post-augmentation. The image data varies in brightness, orientation, and structural features, providing a realistic approximation of clinical noise. Furthermore, class weights were integrated into the categorical cross-entropy loss function to correct for class imbalance during model training. While the dataset lacks pixel-wise segmentation masks, its clean labeling and simplicity make it suitable for testing binary classification pipelines, especially in low-resource or real-time medical diagnostic settings. Its accessibility and well-defined structure also allow easy integration into custom deep learning frameworks for fast experimentation.

### 3.2. Preprocessing

Preprocessing is a commonly used method to improve the quality of input data that arrives to streamline future processing. It is required in this case since the images for the MRI were collected from many artifact-containing modes. As a result, numerous Techniques for processing images are implemented to increase the contrast. Denoising and high-resolution images were created using the Iterative weighted-mean filter (IWMF), which also rectifies the noise and class imbalance issues.

#### 3.2.1. Iterative Weighted-Mean Filter (IWMF).

For improving the image quality and reducing the noise present in the images, different filters are applied in the medical image processing. Adaptive Frequency Median Filter (AFMF) integrates both the standard median filter and mean filter to find a more accurate value of each pixel of the noisy image. However, the computation cost of the AFMF is so high that it limits the performance [39]. The Distribution-based Adaptive Median Filtering (DAMF) technique is used in existing methods to eliminate the additive noise from the input image. However, the DAMF degrades the image quality, specifically when handling the non-impulsive noise, which affects the performance [40]. Discrete Wavelet Transform (DWT) is utilized for decomposing images into different frequency components to improve the image quality for further analysis. Nevertheless, the DWT introduces the artifacts, and its denoising performance is found to be suboptimal [41]. However, the proposed method addresses the above drawbacks with the application of the IWMF filtration method that may smooth images and minimize noise while keeping edges and key features. It is a weighted-mean filter version in which the filter’s coefficients are repeatedly adjusted according to the current filtered image. IWMF is very beneficial when the noise level is excessive and a regular weighted-mean filter is insufficient. The iterative nature of the IWMF approach aids in better adapting to the input image statistics and providing more precise filtering, blurring, denoising, and bias field-effect modifications. Bias field variations in input images refer to spatially varied intensity non-uniformity that can occur for a variety of causes, including variances in scanner sensitivity, imaging equipment characteristics, or patient anatomy [42]. A pixel g in an image I,letI(g) represents its pixel value, and represents its noise recognition matrix. The noise recognition matrix R is expressed as follows:


$$R(g)=\{\;\;\;\begin{array}{c} {}*20c0,0<I(g)<255\\ 1,I(g)=0orI(g)=255 \end{array}$$ (1)

where R(g) =0 indicates the “noise-free pixel” and R(g)=1 indicates the “noise pixel”.

Denoising is performed using a decision-based weighted mean filter with an adjustable window at this step. In the noise detection step, the pixels labeled noise-free (R(g) = 1) stay intact, while the noises (R(g) = 0) are substituted by the regained intensity. The procedure of noise elimination is as follows.

Replacing the intensity of noisy pixels with the previously processed pixel or the mean of processed pixels in the neighborhood results in a streaking effect or artifacts in the restored image. Such issues can be resolved by employing an iterative filter to generate higher-quality photos. The iterative approach for each pixel g in image I is as follows.

1. For each pixel g with R(g) = 1, process g by the method proposed in stage 2.
2. If R is not a zero matrix, repeat 1 until R exists, but use the latest reconstructed image as the input image. Otherwise, leave it alone. If all of the pixels in the image are noisy, the operation should be terminated.

### 3.3. Data augmentation

**Fig 2:**
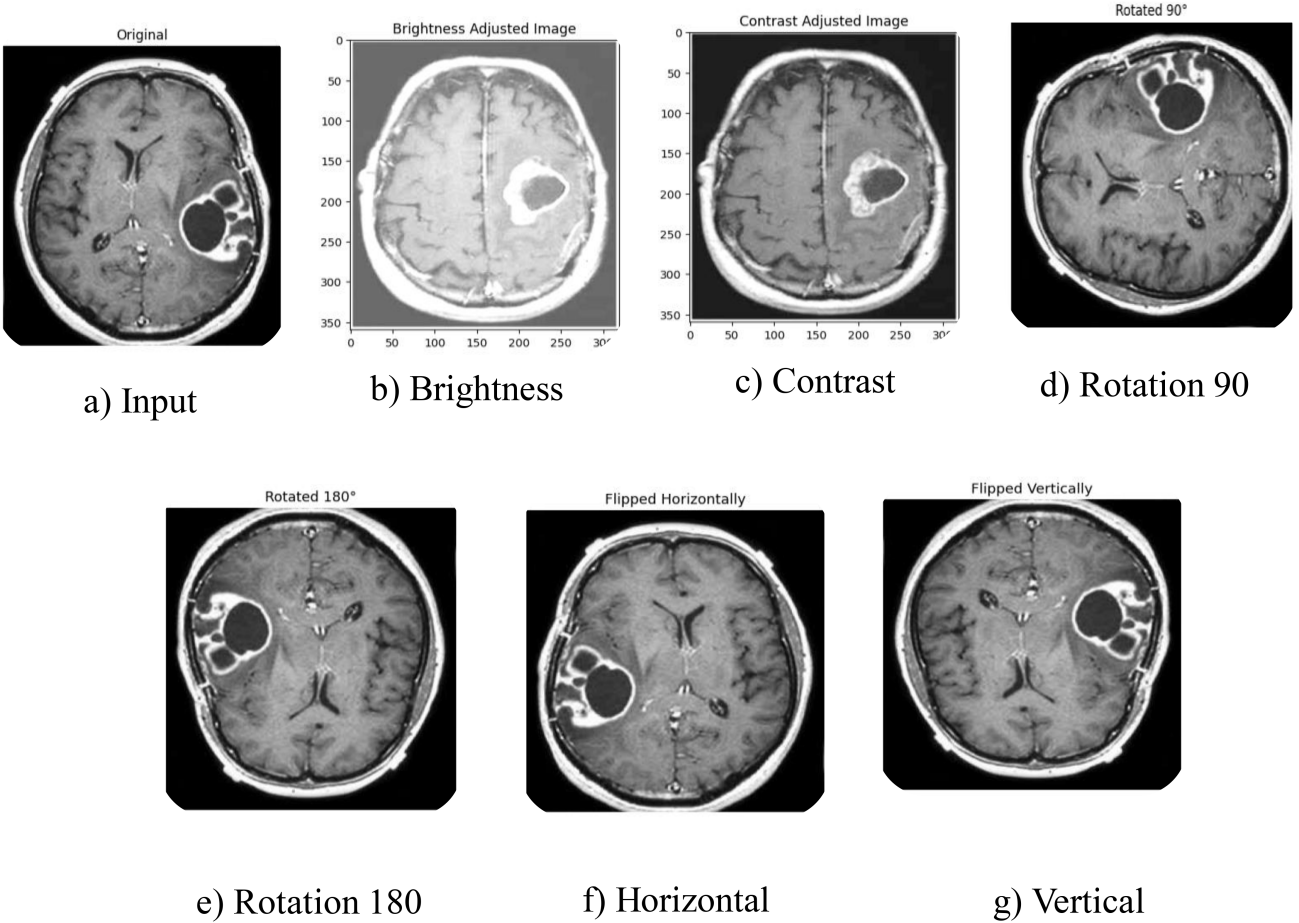
Data Augmentation illustration.

**Fig 3:**
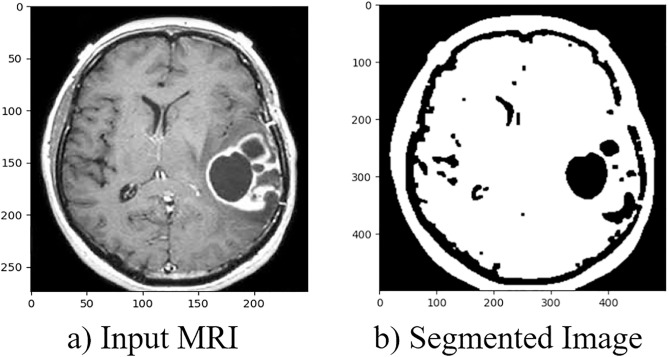
Segmentation Process using MRI. **a)** Input MRI. **b)** Segmented Image.

The preprocessed data is applied to the data augmentation techniques. DL methods require enormous volumes of labeled information for training. Regrettably, the annotated medical image data collection is insufficiently large, which creates issues during training, particularly when employing DL approaches. In the proposed method, data augmentation significantly reduces the class imbalance arising in the datasets. Specifically, the data augmentation increases the number of samples in the minority class, which in turn makes the model less biased towards the majority class. The technique of data augmentation, on the other hand, overcomes this problem arising from expanding the amount of training data accessible [43]. It also helps with overfitting resolution and increases the model’s generalization capabilities during training. As a result, the process of augmentation is used to improve findings on a modest data set. There are several methods for enhancing data, such as Stochastic Brightness Contrast, rotation, and flipping, which are utilized to help the structure understand the changes that occur while training. As shown in Table 2, the subsequent parameters are used throughout the augmentation process. In addition, Figs 2 and 3 show several illustrative images.

**Table 2:** Data augmentation Parameters.

Sl.No	Parameters	Value
1	Vertical Flip	True
2	Horizontal Flip	True
3	Random Brightness Contrast	True
4	Rotation Range	90,180

### 3.4. Segmentation

Image segmentation is a crucial component of numerous visual comprehension systems. It requires separating an image into many parts or items. Segmentation is vital in many applications, including medical image analysis. SqueezeNet segmentation is used to delineate the brain tumor regions from the preprocessed MRI image.

#### 3.4.1. SqueezeNet segmentation.

SqueezeNet serves as the foundation for our encoder-decoder architecture. The encoder is based on the original SqueezeNet design, while the decoder is symmetrically constructed by inverting the downsampling operations of SegNet into corresponding upsampling layers. This symmetric structure enables effective feature reconstruction while maintaining a lightweight model footprint [44]. To adapt SqueezeNet for image segmentation, add an encoder-decoder component to the design. The network’s encoder conducts down-sampling and collects the input image’s high-level characteristics. The decoder element samples the characteristics from the top down to create a segmentation mask. Let’s get to the encoder. It consists of a first convolutional layer composed of filter kernels of stride 2 with size 3 and no padding. This was done in SqueezeNet to achieve a large susceptibility field in the network’s first layer. SqueezeNet was specifically chosen for segmentation in the proposed model due to its lightweight architecture and high computational efficiency, which make it particularly suitable for processing high-resolution medical images like brain MRIs with limited hardware resources. SqueezeNet achieves AlexNet-level accuracy with significantly fewer parameters by using “fire modules” that compress the model without sacrificing performance. This efficiency enables fast and accurate segmentation of brain tissues, which is crucial in isolating tumor regions before classification. While it might seem advantageous to use a single architecture such as Inception for both segmentation and classification, doing so would compromise either speed or specialization. Inception networks are deeper and more computationally intensive, making them more suitable for high-level feature extraction and classification rather than pixel-wise segmentation. Using separate models allows each to focus on its strengths: SqueezeNet handles detailed, efficient segmentation, while Inception V3 combined with BiLSTM excels in learning complex patterns for classification. This modular approach enhances overall system performance, reduces computational burden, and allows fine-tuning of each stage independently for better accuracy and adaptability.

The Fire module is a novel feature of the SqueezeNet image segmentation architecture; it is made up of a squeeze convolution layer (1x1 filters) that reduces parameters before feeding into a combination of two expand layers: one of 1x1 convolution filters and another of 3x3 convolution filters that reduces the number of input channels to 3x3 filters. So we used these fire modules to remove SqueezeNet’s average pooling layer, which was the activation value for object classes. SegNet, which eliminates all entirely linked layers, affected it. The decoder is our proposed approach for inverting the fire and convolutional layers of the SqueezeNet architecture. To begin, we may use a further convolutional layer to invert conv10 for the final convolutional layer form, which is what we have done. In order to create an inverse Fire module, we created a DFire module by concatenating the expand module of SqueezeNet with a squeeze module. Finally, we were motivated to invert the initial layer of convolution (conv1) by H Noh’s work on DeconvNet, who discovered that the deconvolution layer may play several functions in computer vision, the most essential of which is their capacity to increase sample and build a big and dense activation map. It was ideal since the first conv1 decoder layer has a stride of 2, which decreases the spatial dimension of the input image. Finally, it is vital to mention that we invert the SegNet downsample by an upsample layer using the notion of sharing max pool indices from down and upsample layers.

### 3.5. Feature extraction and classification based on Inception V3 enabled deep bidirectional long short term memory network

In this research, the proposed IV3TM model effectively captures the most informative features associated with brain tumors and classifies the different types of brain tumors, including the pituitary, meningioma, and glioma. In the following sections, the overview of the Inception V3 and DBLSTM is provided before discussing the details of the proposed IV3TM model. In the proposed model, especially Inception V3, offers distinct advantages for brain tumor classification due to its advanced architecture designed to capture diverse spatial features at multiple scales. Unlike traditional CNNs that apply fixed-size convolution filters, Inception modules process input data using parallel filters of varying sizes (1 × 1, 3 × 3, 5 × 5), allowing the model to recognize fine-grained textures as well as larger structural abnormalities in MRI scans. This is particularly useful in brain tumor classification, where tumor shape, size, and location can vary widely. Additionally, Inception V3 incorporates factorized convolutions and auxiliary classifiers that help reduce computational complexity and mitigate overfitting—an important concern in medical datasets that are often limited in size. Its deep structure is optimized with batch normalization and regularization techniques that stabilize training and reduce the vanishing gradient problem. When combined with DBLSTM in the proposed hybrid model, Inception V3 efficiently handles spatial feature extraction, complementing the temporal sequence modeling of DBLSTM, thus improving the classification accuracy of brain tumors from segmented MRI regions.

**Inception V3 Module:** The brain tumor characteristics are automatically extracted after image segmentation using the Inception-v3-based transfer learning model [45]. GoogleNet (the inception model) was introduced in 2014. InceptionV3 is one of 42 layers with 24 million parameters in the Inception family. It made improvements to the Inception module to improve ImageNet accuracy. The number of parameters was lowered as a result of the extra factorization without a compromise in network efficiency. The network was one of the first to use batch normalization on its layers. Instead of a large convolution layer, the model utilizes two or three layers of small convolutional layers built around factorized convolution processes, which reduces the parameters without reducing the model’s performance. For this factorization process, the 42-layer Inception-v3 simulation with fewer parameters outperforms the VGGNet. Furthermore, the default weights are applied for this model. This method includes a number of filters for detecting basic features, which are particularly useful for extraction problems. As a result, the photos are twisted in order to extract the relevant attributes.

The NIN structure was created by one of the Google Inception Nets, InceptionV3, which uses Inception modules piled over one another to create a vast network. The structure shows the input size of each segment beneath each part. Two convolution layers, each having a patch size of 3 × 3, and two distinct steps, the first being 2 and the second being 1, were used to generate the module. To double the module’s size, a convolutional padding layer is inserted after the convolutional layers. The 5 × 5 layers of the Inception paradigm were divided into several 3 × 3 and ReLU levels to speed up processing. While the average pooling technique extracts features, the max-pooling approach extracts essential features like edges.

For every sample used for training in Inception-v3, a probability of each label m∈{1,....,m} is calculated by


P(m/ms\nulldelimiterspaces)=exp(ym)∑\nolimitsikexp(yi) (2)

Y stands for the non-normalized log probability. The distribution of ground truth across labels ∑\nolimitsmG(m/ms\nulldelimiterspaces)=1 was normalized. Loss in this system was caused by cross-entropy.


$$F=\sum_{m=1}^{m}\log\left(o(M)\right)G\left(m\right)$$ (3)

Since the cross-entropy loss can be distinguished for log its $y_{k}$, it may be used for gradient training with the DL approach utilizing gradients of the fundamental type \raise0.7ex\({\partial C\)/∂C∂yk\nulldelimiterspace\lower0.7ex\({\partial yk\)}} restricted between −1 and 1.

The log-probability of the right label is often highest while the cross-entropy is minimized. It can therefore lead to issues with overfitting, with the label distributions p(k/kz\nulldelimiterspacez)=∂k,z being switched for training. Inception-v3 employed a smooth function ∈ to analyze distributions among labels during training instances v(k).


G′(m/ms\nulldelimiterspaces)=(1−ε)∂m,s+εv(m) (4)

This is an amalgam of the stable distribution v(k) with weights ∈ and the basic g(m/ms\nulldelimiterspaces) distribution with weights 1−∈. To a uniform distribution v(m)=\raise0.7ex1/1m\nulldelimiterspace\lower0.7exm, the label-smoothing regularization was applied, yielding


g′(m/ms\nulldelimiterspaces)=(1−∈)∂m,s+εm (5)

This could also be considered cross-entropy because,


$$D\left(g^{\prime },X\right)=-\sum_{m=1}^{m}\log\left(x\left(m\right)\right){g^{\prime }}^{\left(m\right)}=\left(1-\in \right)D\left(g^{\prime },x\right)+\in D\left(g^{\prime },x\right)+\in D\left(v,x\right)$$ (6)

On the activation layer, there are several activation features, including sigmoid, ReLU, and softmax. Its goal was to incorporate nonlinear elements to help the situation; it had to be nonlinear as a result. The activation function of the sigmoid function is denoted by,


$$j\left(k\right)=\frac{\text{1}}{1+\ell^{-k}}$$ (7)

The following is a description of ReLU’s activation function:


$$j\left(k\right)=\{\;\;\;\begin{array}{c} {}*20c0,k\leq 0\\ k,k>0 \end{array}$$ (8)

In the following equations, the activation unit and input of the activation unit x are f(x). The components of a convolution are subjected to a nonlinear function called the activation function, comparable to sigmoid or ReLU. The computational issues of a CNN were alleviated when the feature maps created by the convolutional layers included one or more pooling layers. To do this, the size of the maps created by the convolutional layers was lowered. The most common techniques are maximum and average pooling. After testing with many pre-trained CNN models, Inception v3 produced the best F1 score for this assignment.

Feature extraction successfully processes several features of MRI brain images, including area, perimeter, circularity, and intensity values. The characteristics used are determined by the individual challenge and the type of imaging data available. The appropriate use of brain characteristics aids in the successful classification of brain tumors and the analysis of MRI images.

**DBLSTM Module:** In the proposed model, the DBLSTM makes use of numerous layers of BiLSTM units. BiLSTM is a Recurrent Neural Network (RNN) frequently used to analyze sequential input such as text or time series. Over time, neural networks have evolved. An RNN is a prominent component of neural networks, with demonstrated a clear aptitude for sequence and time processing. The LSTM model is a memory cell-centered reworking of the RNN model. Because of the use of specially constructed memory cell units, LSTM outperforms RNN in terms of utilizing and storing data over lengthy intervals of time. Each memory block consists of three gate components: Each of the input, output, and forget gates is able to write, read, and restore the cell’s functionality. For extremely long input sequences, forget gates in particular are critical. Although LSTM has a longer access time to context, both LSTM and RNN are able to utilize information from the past context; they are unable to obtain data from future contexts. As with pattern prediction, extending this to use data in both directions is more useful [46].

**Fig 4:**
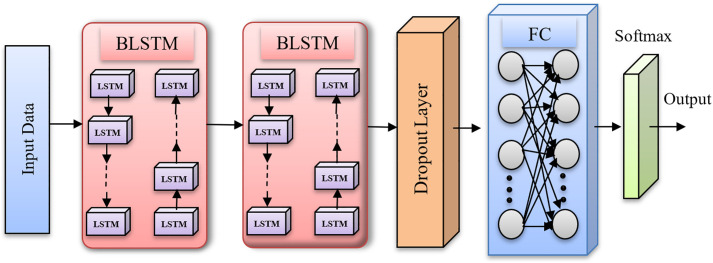
DBLSTM diagram.

A BiLSTM’s fundamental structure is made up of two LSTMs, one analyzing the input sequence forward and the other backward. Because the network is bidirectional, the model records dependencies from both the past and the future, allowing for a better comprehension and representation of the sequential data. The structure of DBLSTM is depicted in Fig 4. As shown in Fig 1, Inputs are passed forward through the Forward Layer for t = 1 to T and the Backward Layer with t = T to 1, and the Forward as well as Backward Layers are subsequently sent forward to the same Output Layer. As a result, the BLSTM may access information in the past and future as well as leverage context over a lengthy period of time. With the deep feed-forward network in mind, additional recurring concealed layers were stacked on top of one another to provide a richer data representation, resulting in the final DBLSTM model utilized in this research.

DBLSTM takes this notion a step further by stacking many levels of BiLSTM units on top of one another. Each layer accepts the preceding layer’s output as input, enabling hierarchical learning and information abstraction. The extra layers allow the network to acquire more complex patterns and representations from sequential input. A DBLSTM training approach involves forward and backward propagation through all levels. The input sequence is processed through the layers during forward propagation, and the output of the final layer is acquired. The gradients are then calculated and updated via each layer during backpropagation to optimize the network’s parameters. The provided network diagram illustrates a Bidirectional Long Short-Term Memory (BLSTM) architecture designed for sequential data classification. The input data, likely a reshaped feature sequence (e.g., from InceptionV3 features), enters the network as a matrix of shape [batch_size, sequence_length, feature_dim], such as [47,64]. This is followed by two stacked BLSTM layers. The first BLSTM layer processes the sequence in both forward and backward directions with, for example, 128 units in each direction, outputting a tensor of shape [sequence_length, 256]. The second BLSTM layer further captures temporal dependencies with fewer units (e.g., 64 per direction), producing an output of [sequence_length, 128]. After sequence modeling, a dropout layer is applied for regularization. The data is then passed through a fully connected (FC) layer, typically reducing the dimensionality to 64 or another suitable value. Finally, the softmax output layer produces the probability distribution across the target classes (e.g., tumor types), with the output dimension matching the number of classification labels. Including specific dimensions at each layer enhances model transparency and reproducibility. Following correct segmentation and feature extraction, the images must be classified based on this phase. Initially, the model will be trained and tested using a combination of the Inception V3 and DBLSM methods, using the RMSE and Accuracy parameters. In the last step, a comparison analysis will be performed to present the results based on the parameters that have been set to evaluate the work’s performance.

Although BiLSTM (Bidirectional Long Short-Term Memory) networks are traditionally used for sequential and time-series data, their application in brain tumor classification is motivated by their ability to model long-range dependencies and contextual relationships across spatial features extracted from medical images. In the proposed hybrid model, after the Inception V3 network extracts rich spatial features from segmented MRI images, these features are treated as a structured sequence, allowing the BiLSTM to analyze them in both forward and backward directions. This bidirectional processing enables the network to capture intricate patterns and contextual cues that may not be evident when considering spatial information in a unidirectional or purely localized manner. Such an approach is particularly beneficial in brain MRI analysis, where the relationships between adjacent tissue regions or segmented slices can provide critical diagnostic information. By leveraging BiLSTM’s memory capabilities and contextual sensitivity, the hybrid model enhances feature representation and improves classification performance, especially in distinguishing subtle differences between tumor types.

In this hybrid approach, Inception V3 acts as the convolutional backbone responsible for extracting deep spatial features from the segmented MRI image of the brain. Instead of flattening these features directly into a dense layer, the output feature maps from Inception V3 are reshaped into a temporal sequence. This sequence is then passed into the DBLSTM, which learns bidirectional contextual relationships across the spatial dimensions. The DBLSTM processes the sequence both forward and backward, enhancing the model’s understanding of spatial dependencies between regions in the image. In the proposed model, the integration of the deep architecture with multiple inception layers facilitates the model to learn hierarchical representations of the input MRI data. Further, the proposed model leverages the DBLSTM module for improving the feature representations from sequential input to analyze the underlying characteristics of the input data and reduces the risk of overfitting.

**Fig 5:**
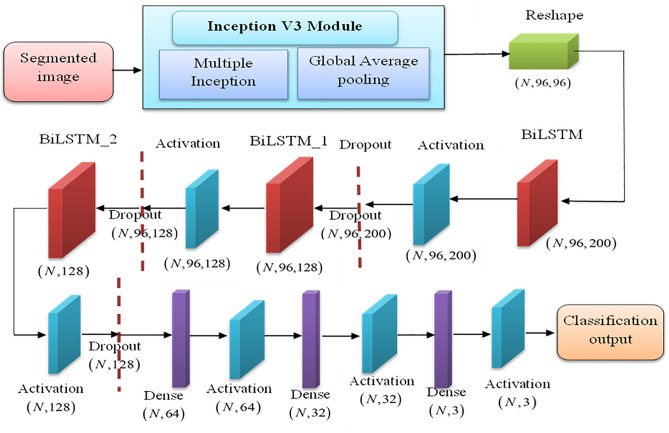
Architecture of the proposed IV3TM Model.

The proposed IV3TM architecture shown in Fig 5 effectively integrates the strengths of both spatial and sequential modeling by combining Inception V3 and DBLSTM in a hybrid deep learning pipeline. The process begins with input MRI images, typically of size 299x299 pixels, which are passed through the Inception V3 backbone. This component is responsible for extracting rich spatial features using its multiple convolutional pathways and global average pooling, yielding a compact 1 × 1 × 2048 feature map. This output is then reshaped into a sequential format (e.g., [47,64]), enabling the DBLSTM layers to process it as a temporal sequence. The two stacked bidirectional LSTM layers capture forward and backward dependencies within the spatial features, enhancing contextual understanding across regions of the brain. The resulting feature representations are passed through a fully connected dense layer with ReLU activation, followed by a softmax layer that performs the final classification into tumor categories such as glioma, meningioma, or no tumor. This hybrid design capitalizes on the powerful feature extraction capabilities of Inception V3 and the contextual modeling strength of DBLSTM, ultimately leading to improved classification performance in complex brain tumor cases.

## 4. Result and discussion

The results obtained with the implementation of the proposed IV3TM model for brain tumor classification are detailed in this section. The performance is examined using various datasets, such as brain tumor datasets from Figshare and MRI images of the brain. Further, the results of segmentation and classification are shown to be superior in terms of precision, F1 score, accuracy, and recall compared to previous approaches. Additionally, the proposed method evaluates the error using the root mean square error (RMSE).

### 4.1. Experimental setup

The experimental setup for the implementation of the proposed IV3TM model is detailed in Tables 3 and 4 as follows,

**Table 3:** Experimental Setup for Hybrid Inception V3–BiLSTM Model.

Component	Details
Platform & Framework	TensorFlow 2.10/ Keras with Python 3.7
Hardware Used	NVIDIA RTX 3090 GPU, 64 GB RAM, Intel i9 Processor
Validation Strategy	90/10 Train-Test Split
BiLSTM Configuration	2 Bidirectional LSTM layers, 256 hidden units each
Activation Function	ReLU (Inception), Tanh/Sigmoid (BiLSTM), Softmax (Final Classification)
Loss Function	Categorical Cross-Entropy
Optimizer	Adam Optimizer
Learning Rate	0.0001 (with ReduceLROnPlateau scheduler)
Batch Size	32
Number of Epochs	500
Regularization	Dropout (0.5 in BiLSTM layers), L2 (0.001) in fully connected layers

**Table 4:** Parameter settings.

		Data Augmentation Parameters
**Model**	DBLSTM	Horizontal = 0.5
**Simulation**	Python	Vertical = 0.5
**Input Image Size**	512 × 512	Random Contrast Brightness = 0.3
**Mini-Batch**	32	Rotate = 90°
**Framework**	Keras	Rotate = 180°

### 4.2. Database description

The Figshare and Brain MRI image collections are where the brain tumor MRI images were found. This dataset’s images have previously been divided into training and testing data. They have a total of 3,064 MRI brain slices from 233 patients (2670 files in the training part and 394 in the testing portion), as well as 98 images of the brain without a tumor and 155 images of tumors in the Brain MRI dataset. There are 394 images in every group (benign or malignant) in the testing set, and each category in the training dataset contains two file formats. As shown in Table 5 dataset distribution in the proposed method.

**Table 5:** Dataset distribution in the proposed method.

Dataset	Number of Images
Brain MRI image dataset	253
Figshare brain tumor dataset	3,064

Denoising the input image during image preprocessing will boost the final image’s quality. The next crucial stage in many processing pipelines is segmentation, which can shorten computation times simply by matching quantitative maps inside the brain or increase the accuracy of segmentations of brain tumors. The parameters for the features were retrieved when the segmentation was finished. It was meant for the output of a neural network with 1,000 hidden units to divide brain cancers into two groups.

**Fig 6:**
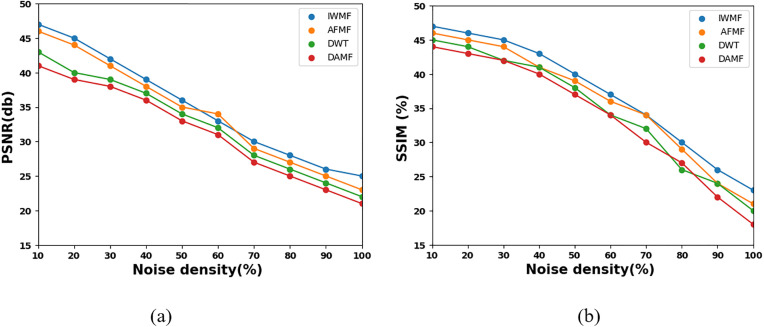
Compare different methods on MRI average values of (a) PSNR and (b) SSIM.

Fig 6 shows the comparison of different methods on MRI average values of (a) PSNR and (b) SSIM. The proposed IWMF outperformed all other filters in terms of noise reduction, determined by the measurements of the Peak Signal-to-Noise Ratio (PSNR) and Structural Similarity Index (SSIM). Although the AFMF and DWT achieved satisfactory results, the DAMF generated the least PSNR and SSIM throughout the majority of noise density levels. PSNR and SSIM results for the AFMF, DWT, and DAMF filters were comparable but less than IWMF values. Fig 9 further demonstrated that for noise intensities ranging from 0.1 to 0.9, the IWMF delivered the highest PSNR and SSIM values, indicating that the IWMF functioned best in terms of image detail retention and image quality.

**Fig 7:**
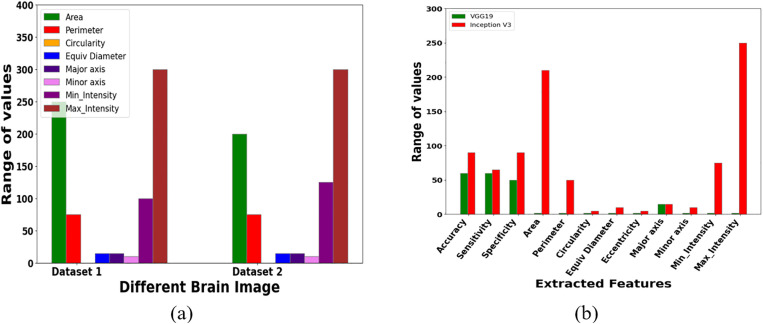
Different Range values of the extracted features using the Brain MRI image dataset and the Figshare brain tumor dataset.

**Fig 8:**
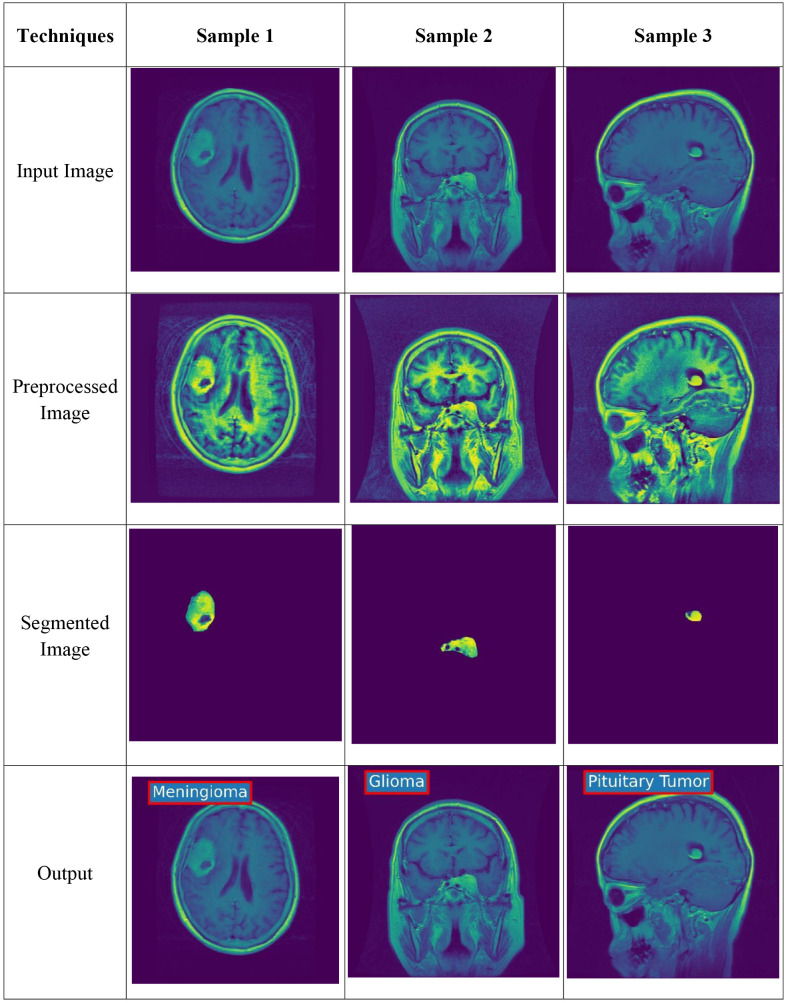
Experimental results obtained using the IV3TM Model for brain tumor classification.

Fig 7(a) shows how to effectively extract various MRI brain image properties such as intensity values, perimeter, area, and circularity. Fig 7(a) depicts the use of brain functions effectively in successfully classifying the brain tumors. Fig 7(a) shows an analysis of the effectiveness of the Inception V3 features. The database’s tumor and non-tumor photos were chosen at random for training and testing.

The graph comparing performance-optimized parameters for VGG19 and Inception V3 is displayed in Fig 7(b). The development of an automatic tumor diagnosis and treatment assessment procedure uses a brain tumor classification approach. From an MRI brain image, the tumor area is thought to be able to determine whether a brain tumor is abnormal. Then, utilizing Sensitivity, Specificity, and Accuracy metrics, the effectiveness of MRI brain tumor identification is evaluated. It has been compared how well VGG19 and Inception V3 perform. Thus, the following Table 2 shows the Experimental Setup for Brain Tumor Classification using a Hybrid Inception V3–BiLSTM Model.

### 4.3. Experimental results

The experimental results obtained with the implantation of the proposed IV3TM Model for brain tumor classification for different datasets are illustrated in Fig 8. Further, the experimental analysis explained with the input image, preprocessed image, segmented image, and the classified output, which demonstrates the effectiveness of the proposed model in classifying the brain tumor.

### 4.3. Performance metrics

The training phase is for learning, while the validation and testing sets are for evaluating performance. Five criteria are used to evaluate categorization tasks: The model’s functioning was assessed using accuracy, precision, F1 score, specificity, and sensitivity. The ROC curve was used in the research study to assess the performance of the classifiers. The genuine positive value is compared against the false positive value over a wide range of values. It shows the classifier’s ability to distinguish between the four classes.

**Accuracy:** In classification tasks, accuracy is given as a percentage simply by dividing the overall number of successfully identified images by the total number of images. It analyses all of the pixels in an image.


$$Accuracy=\frac{TN+TP}{FN+TP+TN+FP}$$ (9)

**Precision:** The fraction of samples that were both actually positive and expected to be positive, compared to the overall sample count projected to be positive.


$$\mathrm{Pr}ecision=\frac{TP}{FP+TP}$$ (10)

**Sensitivity (Recall):** is the percentage of positive samples in all samples.


$$Sensitivity(Re\mathrm{\backslash nolimits}call)=\frac{TP}{TP+FN}$$ (11)

**Specificity:** The percentage of negative samples predicted out of all negative cases is the specificity of the real negative value.


$$Specificity=\frac{TN}{FP+TN}$$ (12)

**F1 Score**-The Harmonic measure of recall and precision.


$$F1-score=2\times \frac{\mathrm{Pr}ecision\times Re\mathrm{\backslash nolimits}call}{\mathrm{Pr}ecision+Re\mathrm{\backslash nolimits}call}$$ (13)

### 4.4. Accuracy and loss curves

**Fig 9:**
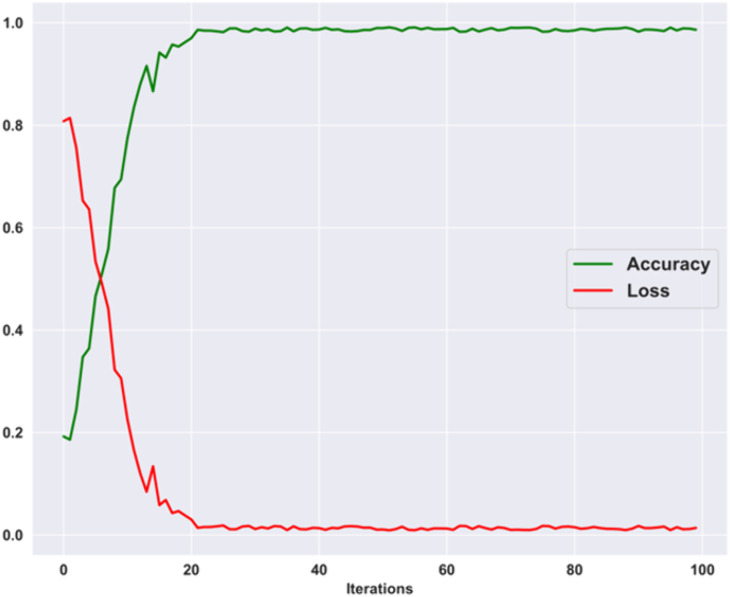
Accuracy and Loss Curve.

Fig 9 displays the accuracy and loss curves obtained with the training process for several iterations. For the proposed model, the accuracy and loss graphs demonstrate that the accuracy of the training steadily increases in a shorter period and reaches a point of stability that reveals the effectiveness of the proposed model. Further, the loss decreases with the increasing number of iterations, highlighting the improved learning ability of the proposed model for classifying the brain tumor instances.

### 4.5. Performance evaluation

The performance of the proposed IV3TM model is evaluated by varying the training epochs for different percentages of training, and the results obtained with the application of the Brain MRI dataset and the Figshare dataset are analyzed in this section.

#### 4.5.1. Performance evaluation with training percentage using brain MRI dataset.

**Fig 10:**
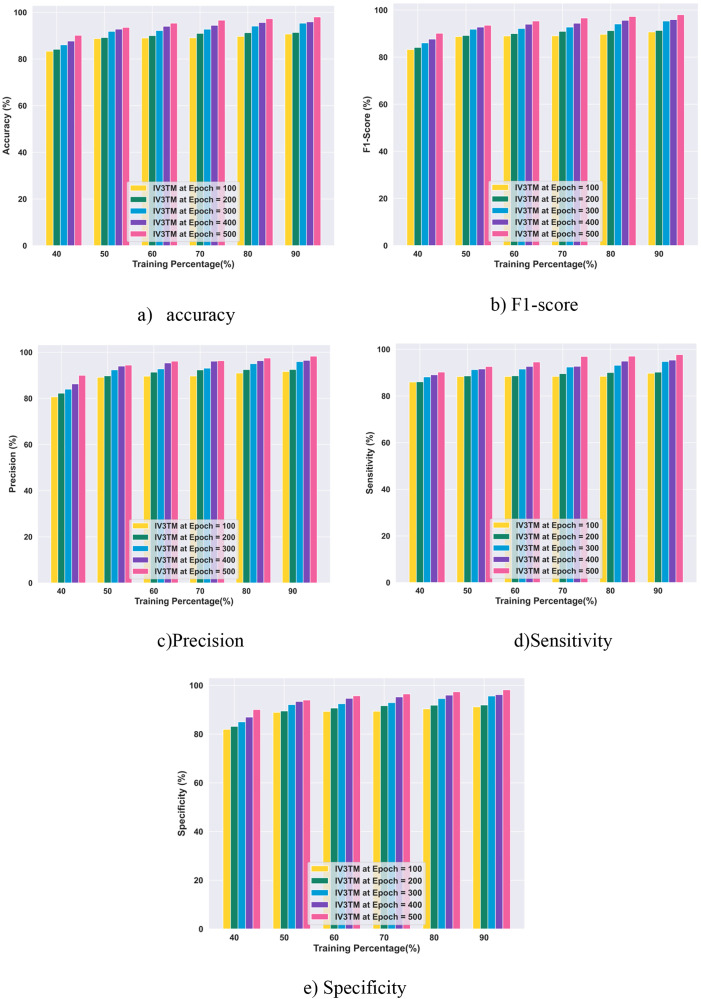
Performance Evaluation with Training Percentage using Brain MRI Dataset.

The performance evaluation of the proposed IV3TM model in terms of training percentage analysis with the Brain MRI dataset is displayed in Fig 10. The results demonstrate that the IV3TM model achieves a high accuracy of 90.71% for epoch 100, 91.36% for epoch 200, 95.36% for epoch 300, 95.94% for epoch 400, and 98.02% for epoch 500.In terms of metric F1-score, the proposed IV3TM model attains the best performance, gaining 90.70% for epoch 100, 91.35% for epoch 200, 95.36% for epoch 300, 95.93% for epoch 400, and 98.02% for epoch 500. Subsequently, the proposed IV3TM model reports the precision values of 91.67%, 92.52%, 95.97%, 96.49%, and 98.30% for the respective epochs 100, 200, 300, 400, and 500. In addition, the proposed IV3TM model achieves superior performance in terms of metric sensitivity, obtaining the values of 89.75%,90.21%,94.76%,95.39%, and 97.74%, for the epochs 100, 200, 300, 400, and 500, respectively. Further, the evaluation results of the proposed IV3TM model for brain tumor classification report the specificity values of 91.19%, 91.93%, 95.66%, 96.22%, and 98.16% for the corresponding epochs of 100, 200, 300, 400, and 500. Moreover, the results obtained with the performance analysis demonstrate that the performance of the proposed IV3TM model improves with increasing number of epochs.

#### 4.5.2. Performance evaluation with training percentage based on the Figshare dataset.

**Fig 11:**
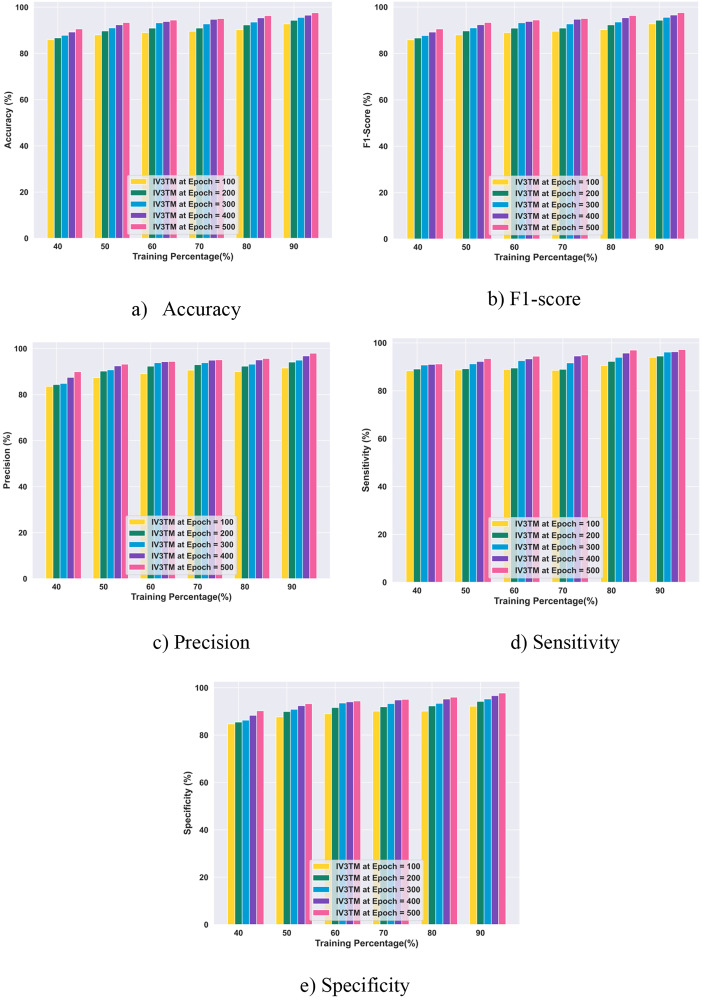
Performance Evaluation with Training Percentage based on the Figshare dataset.

The results obtained with the performance evaluation of the proposed IV3TM model for different percentages of training on the Figshare dataset are portrayed in Fig 11. From the performance evaluation, the proposed IV3TM achieves the accuracy of 92.76% for epoch 100, 94.32% for epoch 200, 95.56% for epoch 300, 96.55% for epoch 400, and 97.59% for epoch 500. Subsequently, the proposed IV3TM model reports the F1-score of 92.75% for epoch 100, 94.32% for epoch 200, 95.56% for epoch 300, 96.55% for epoch 400, and 97.59% for epoch 500. Similarly, the proposed IV3TM model achieves the precision values of 91.58%, 94.15%, 94.98%, 96.80%, and 97.98% for the corresponding epochs of 100, 200, 300, 400, and 500. Meanwhile, the proposed IV3TM model demonstrates superior performance for sensitivity, obtaining the values of 93.94%, 94.49%, 96.14%,96.29%, and 97.20%, for the epochs 100, 200, 300, 400, and 500, respectively. Besides, the proposed IV3TM model obtains high specificity in classifying the tumor instances, achieving 92.17%, 94.23%, 95.27%, 96.68%, and 97.78% for the corresponding epochs of 100, 200, 300, 400, and 500. Overall, the findings of the research reveal the effectiveness of the proposed IV3TM model in improving the brain tumor classification.

### 4.6. Comparative evaluation

In this section, the results obtained with the comparative evaluation of the proposed IV3TM model with other existing models, such as CNN-SVM [28], VGG-SCNet [48], GCNN [49], IACO-ResNet [50], Caps-VGGNet [51], and CJHBA-DRN [52] for classifying the brain tumor instances are analyzed in detail. The effectiveness of the proposed model over other competing methods is evaluated in terms of training percentage analysis for both the Brain MRI dataset and the Figshare dataset.

#### 4.6.1. Comparative evaluation with training percentage based on the brain MRI dataset.

**Fig 12:**
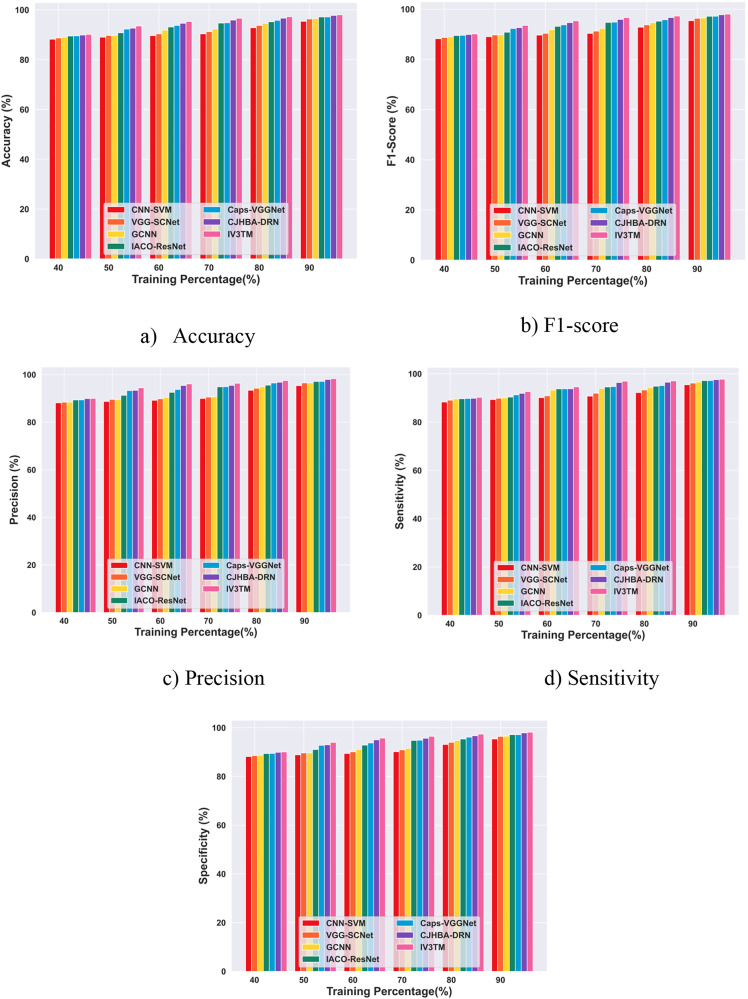
Comparative Evaluation with Training Percentage based on the Brain MRI dataset.

The comparative analysis results for the proposed IV3TM model with other existing techniques using the Brain MRI dataset are presented in Fig 12. The results obtained with the comparison indicate that the proposed model reported a high accuracy of 98.02%, indicating the performance improvement of 2.66% over CNN-SVM, 1.71% over VGG-SCNet, 1.49% over GCNN, 0.87% over IACO-ResNet, 0.86% over Caps-VGGNet, and 0.25% over CJHBA-DRN on 90% of training. Notably, the proposed IV3TM model achieves a high F1-score of 98.02%, outperforming the other existing techniques with a significant improvement of 2.66% against CNN-SVM, 1.71% against VGG-SCNet, 1.50% against GCNN, 0.88% against IACO-ResNet, 0.86% against Caps-VGGNet, and 0.26% against CJHBA-DRN. Similarly, the proposed IV3TM model demonstrates a high precision of 98.30%, significantly exceeding the other existing techniques with the relative improvement of 3.00% over CNN-SVM, 1.81% over VGG-SCNet, 1.80% over GCNN, 1.18% over IACO-ResNet, 1.17% over Caps-VGGNet, and 0.33% over CJHBA-DRN. For metric sensitivity, the proposed IV3TM model reports a high performance of 97.74%, surpassing the competing method CNN-SVM by 2.33%, VGG-SCNet by 1.61%, GCNN by 1.19%, IACO-ResNet by 0.57%, Caps-VGGNet by 0.55%, and 0.18% over CJHBA-DRN. Besides, the proposed IV3TM model attains a high specificity of 98.16%, demonstrating the substantial improvement of 2.83% compared to CNN-SVM, 1.76% compared to VGG-SCNet, 1.65% compared to GCNN, 1.03% compared to IACO-ResNet, 1.02% compared to Caps-VGGNet, and 0.29% compared to CJHBA-DRN. Moreover, the proposed model, combining the Inception v3 and DBLSTM, captures both the fine-grained and high-level features to analyze the complex patterns, contributing to its superior performance Fig 12. Comparative Evaluation with Training Percentage based on the Brain MRI dataset.

#### 4.6.2. Comparative evaluation with training percentage based on the Figshare dataset.

**Fig 13:**
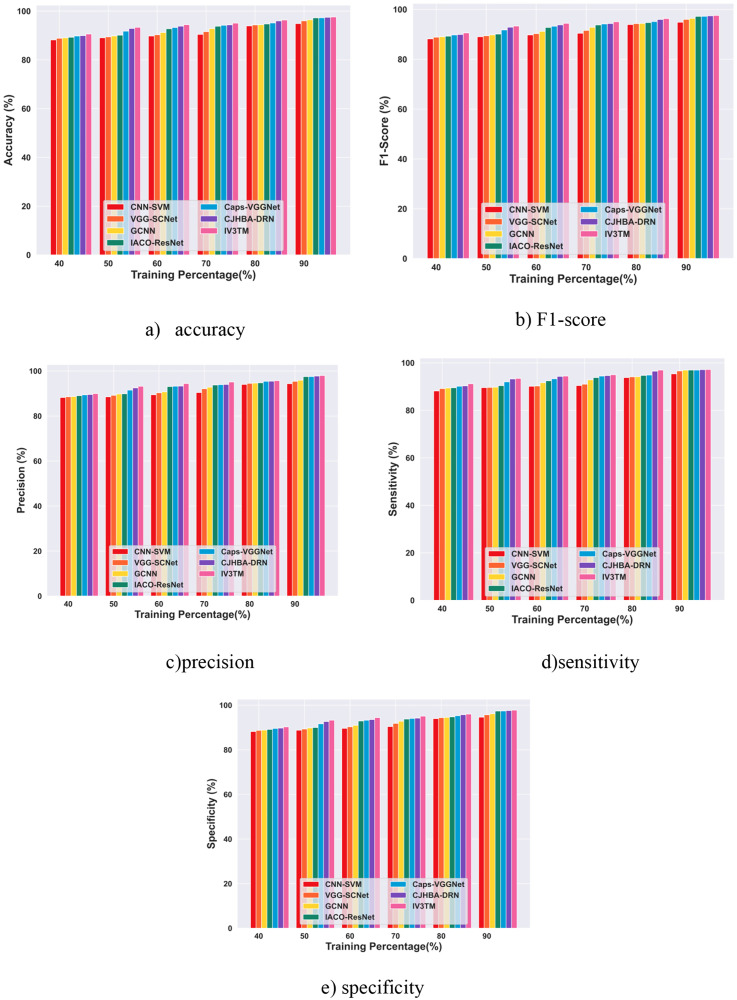
Comparative Evaluation with Training Percentage Analysis on the Figshare dataset.

The results obtained with the comparison of the proposed IV3TM model with other competing methods using the Figshare dataset are presented in Fig 13. From the comparative evaluation, the proposed IV3TM model gained a high accuracy of 97.58% for 90% of training, surpassing the existing CNN-SVM by 2.79%, VGG-SCNet by 1.63%, GCNN by 1.19%, IACO-ResNet by 0.36%, Caps-VGGNet by 0.34%, and CJHBA-DRN by 0.14%. Subsequently, the proposed IV3TM model attains a high F1-score of 97.58%, exceeding the other existing methods with a substantial improvement of 2.78% against CNN-SVM, 1.62% against VGG-SCNet, 1.19% against GCNN, 0.36% against IACO-ResNet, 0.33% against Caps-VGGNet, and 0.14% against CJHBA-DRN. In addition, the proposed IV3TM model achieves a high precision of 97.97%, significantly outperforming the other existing methods with the relative improvement of 3.68% over CNN-SVM, 2.63% over VGG-SCNet, 2.13% over GCNN, 0.49% over IACO-ResNet, 0.44% over Caps-VGGNet, and 0.23% over CJHBA-DRN. Further, the proposed IV3TM model gains a high sensitivity of 97.19%, exceeding the conventional methods with a significant improvement of 1.89% over CNN-SVM, 0.61% over VGG-SCNet, 0.25% over GCNN, 0.24% over IACO-ResNet, 0.23% over Caps-VGGNet, and 0.05% over CJHBA-DRN. With 90% of training, the proposed IV3TM model reported a high specificity of 97.78%, representing the relative improvement of 3.23% against CNN-SVM, 2.13% against VGG-SCNet, 1.66% against GCNN, 0.42% against IACO-ResNet, 0.39% against Caps-VGGNet, and 0.19% against CJHBA-DRN. Overall, the proposed model integrating the Inception v3 and DBLSTM captures the subtle variations in the input data and effectively analyzes the complex patterns, resulting in superior performance compared to other existing methods Fig 13. Comparative Evaluation with Training Percentage Analysis on the Figshare dataset.

### 4.7. Comparative discussion

Tables 6 and 7 show the comparison of results obtained with the proposed method with existing methods, including the CNN-SVM, VGG-SCNet, GCNN, IACO-ResNet, Caps-VGGNet, and CJHBA-DRN. Despite their effectiveness, the existing methods exhibit specific challenges that limit their performance. Specifically, the VGG-SCNet method has a limited number of images, affecting the generalization performance. Further, the VGG-SCNet method only classified the brain tumors from 2D data, limiting their overall applicability [48]. In addition, the GCNN structure is limited for reuse to identify a modest number of images, forming the major drawback of the method [49]. Further, the interpretability of the Caps-VGGNet model is limited due to the complex nature of CapsNet and VGGNet architectures [51]. Besides, the CJHBA-DRN method requires integrating more features to improve the overall performance [52]. However, the proposed approach addressed the drawbacks in the existing methods with the application of the IV3TM model that captures the most subtle features contributing to improved classification accuracy and shows improved generalization capabilities with the utilization of SqueezeNet Segmentation and outperforms the other existing techniques.

**Table 6:** Comparative discussion of the proposed IV3TM model based on the Brain MRI dataset.

Methods	Accuracy (%)	F1-score (%)	Precision (%)	Sensitivity (%)	Specificity (%)
CNN-SVM	95.41	95.41	95.36	95.47	95.39
VGG-SCNet	96.35	96.34	96.52	96.17	96.44
GCNN	96.56	96.55	96.54	96.58	96.55
IACO-ResNet	97.17	97.17	97.14	97.19	97.15
Caps-VGGNet	97.18	97.17	97.15	97.20	97.17
CJHBA-DRN	97.77	97.77	97.98	97.57	97.87
**Proposed IV3TM**	**98.02**	**98.02**	**98.30**	**97.74**	**98.16**

**Table 7:** Comparative discussion of the proposed IV3TM model based on the Figshare dataset.

Methods	Accuracy (%)	F1-score (%)	Precision (%)	Sensitivity (%)	Specificity (%)
CNN-SVM	94.87	94.86	94.37	95.36	94.62
VGG-SCNet	96.00	95.99	95.40	96.60	95.70
GCNN	96.43	96.42	95.90	96.95	96.16
IACO-ResNet	97.23	97.23	97.50	96.97	97.37
Caps-VGGNet	97.26	97.25	97.54	96.97	97.40
CJHBA-DRN	97.45	97.44	97.75	97.15	97.60
**Proposed IV3TM**	**97.59**	**97.58**	**97.98**	**97.20**	**97.78**

### 4.8. Computation complexity analysis

**Fig 14:**
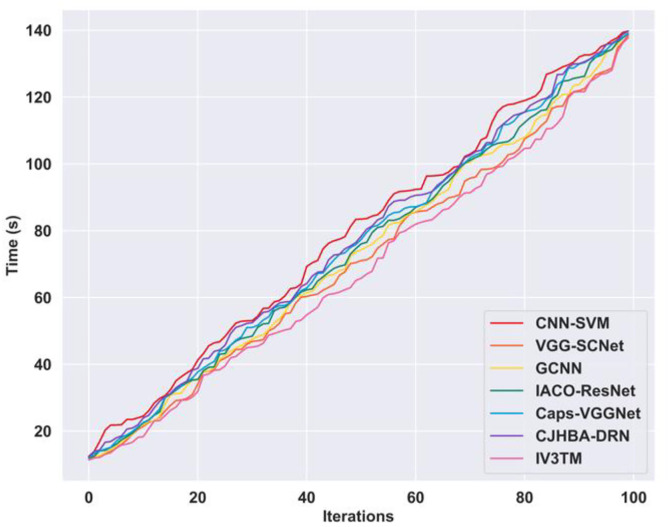
Computation complexity analysis.

Computation complexity analysis is carried out to assess the computational capability of the proposed IV3TM model over other competing methods in brain tumor classification. From the analysis, the proposed IV3TM model minimized the computation time to 137.79s for iteration 100, representing the high computation capability of the proposed method over other existing techniques. As a result, the proposed IV3TM model reported the significant time difference of 1.98s over CNN-SVM,0.67s over VGG-SCNet,1.09s over GCNN,1.12s over IACO-ResNet,1.28s over Caps-VGGNet, and 1.95s over CJHBA-DRN. More specifically, the improved segmentation using SqueezeNet reduces the training time and ensures that the model converges faster with fewer iterations. Moreover, the proposed method reduces the overall computational complexity, making the method highly efficient for real-world applications. Fig 14 depicts the computational complexity analysis of the proposed method and other competing methods.

### 4.9. Statistical analysis

Statistical significance testing is carried out to evaluate the robustness of the reported results in terms of different statistical measures. For evaluating the results, the statistical measures including the mean, variance, and Standard deviation (STD) are utilized for assessing the results reported in terms of metrics, Accuracy, precision, sensitivity, specificity, and F1score. The results of statistical analysis obtained with the Brain MRI dataset and the Figshare dataset are portrayed in Tables 8 and 9, respectively. Overall, the results obtained with the statistical analysis reveal that the proposed IV3TM framework outperforms other competing techniques utilized for the brain tumor classification.

**Table 8:** Statistical Analysis with the Brain MRI dataset.

Methods/ Metrics		CNN-SVM	VGG-SCNet	GCNN	IACO-ResNet	Caps-VGGNet	CJHBA-DRN	IV3TM
Accuracy	Mean	90.92	91.70	92.35	93.43	93.91	94.58	95.16
	Variance	6.10	6.76	6.75	6.85	6.07	7.01	7.13
	STD	2.47	2.60	2.60	2.62	2.46	2.65	2.67
F1-score	Mean	90.92	91.70	92.34	93.42	93.91	94.58	95.16
	Variance	6.10	6.77	6.76	6.85	6.08	7.01	7.14
	STD	2.47	2.60	2.60	2.62	2.47	2.65	2.67
Precision	Mean	90.80	91.52	91.77	93.46	94.16	94.83	95.45
	Variance	7.07	8.30	8.56	7.07	6.47	6.79	7.37
	STD	2.66	2.88	2.93	2.66	2.54	2.60	2.71
Sensitivity	Mean	91.04	91.88	92.94	93.39	93.66	94.34	94.87
	Variance	5.38	5.56	5.96	6.88	6.05	7.62	7.27
	STD	2.32	2.36	2.44	2.62	2.46	2.76	2.70
Specificity	Mean	90.86	91.61	92.06	93.44	94.03	94.70	95.31
	Variance	6.55	7.49	7.54	6.93	6.23	6.85	7.21
	STD	2.56	2.74	2.75	2.63	2.50	2.62	2.68

**Table 9:** Statistical Analysis with the Figshare dataset.

Methods/ Metrics		CNN-SVM	VGG-SCNet	GCNN	IACO-ResNet	Caps-VGGNet	CJHBA-DRN	IV3TM
Accuracy	Mean	91.04	91.74	92.28	92.99	93.57	94.06	94.56
	Variance	6.16	6.76	6.64	7.28	5.70	5.62	4.97
	STD	2.48	2.60	2.58	2.70	2.39	2.37	2.23
F1-score	Mean	91.04	91.74	92.28	92.99	93.56	94.06	94.55
	Variance	6.16	6.75	6.64	7.28	5.70	5.62	4.98
	STD	2.48	2.60	2.58	2.70	2.39	2.37	2.23
Precision	Mean	90.83	91.68	92.09	93.00	93.51	93.77	94.40
	Variance	6.17	6.63	6.71	8.24	6.85	6.42	6.01
	STD	2.48	2.57	2.59	2.87	2.62	2.53	2.45
Sensitivity	Mean	91.25	91.81	92.47	92.98	93.62	94.36	94.71
	Variance	6.28	7.15	6.73	6.41	4.68	4.97	4.22
	STD	2.51	2.67	2.59	2.53	2.16	2.23	2.05
Specificity	Mean	90.93	91.71	92.19	93.00	93.54	93.92	94.48
	Variance	6.15	6.66	6.65	7.75	6.25	6.00	5.46
	STD	2.48	2.58	2.58	2.78	2.50	2.45	2.34

### 4.10. Receiver Operating Characteristic (ROC)

**Fig 15:**
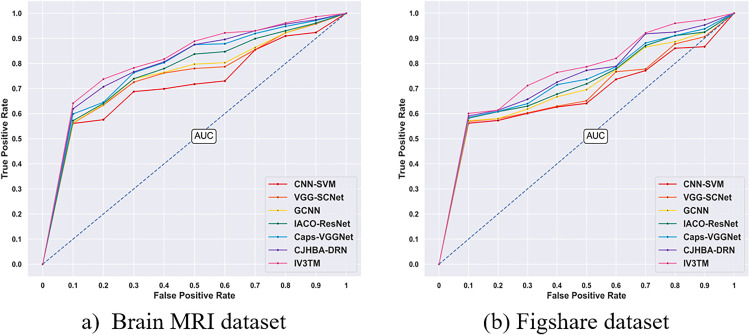
ROC analysis. **a)** Brain MRI dataset. **(b)** Figshare dataset.

ROC Analysis is conducted for assessing the performance of the proposed model in correctly classifying the types of brain tumor instances. Specifically, the ROC curve is plotted against True Positive Rate (TPR) or sensitivity and False Positive Rate (FPR) or error rate in classifying the brain tumor instances. From the ROC curve with the Brain MRI dataset, the competing methods, including the CNN-SVM, VGG-SCNet, GCNN, IACO-ResNet, Caps-VGGNet, and CJHBA-DRN, achieved the TPR values of 0.92, 0.95, 0.95, 0.96, 0.97, and 0.97, respectively. Contrastingly, the proposed IV3TM model reported a high sensitivity of 0.99, indicating the model’s high capability in brain tumor classification. Similarly, the ROC analysis with the Figshare dataset shows that the proposed method obtains a high sensitivity of 0.97, indicating the improved performance of the IV3TM model in classifying the tumor instances. On the other hand, the competing methods such as CNN-SVM, VGG-SCNet, GCNN, IACO-ResNet, Caps-VGGNet, and CJHBA-DRN report the TPR values of 0.87, 0.91,0.92,0.92,0.94, and 0.95, respectively. Moreover, the ROC curve demonstrates the effectiveness of the proposed IV3TM model to discriminate between true positives and false positives. Overall, the ROC analysis shown in Fig 15 reveals the significance of the IV3TM model in improving the brain tumor classification.

### 4.11. Confusion matrix

**Fig 16:**
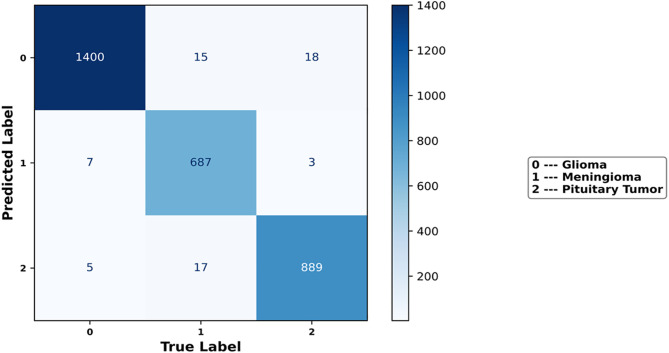
Confusion Matrix.

The predictions from the classification problem are compiled in the Confusion Matrix. Specifically, the confusion matrix is used for evaluating the proposed model in correctly classifying the tumor instances present in the dataset. Different assessment metrics, including F1 score, recall, precision, and accuracy, can be constructed using the data from the confusion matrix to evaluate the effectiveness of a classification model. These metrics assist in assessing the model’s overall performance by giving information about how well it can classify the instances of various classes. Overall, the confusion matrix in Fig 16 shows that the proposed IV3TM model correctly classifies the 1400 glioma instances, 687 meningioma instances, and 889 pituitary tumor instances. Further, the proposed IV3TM model shows a minimum number of misclassifications and has significant implications for developing more precise medical diagnosis systems.

### 4.12. Ablation study

**Fig 17:**
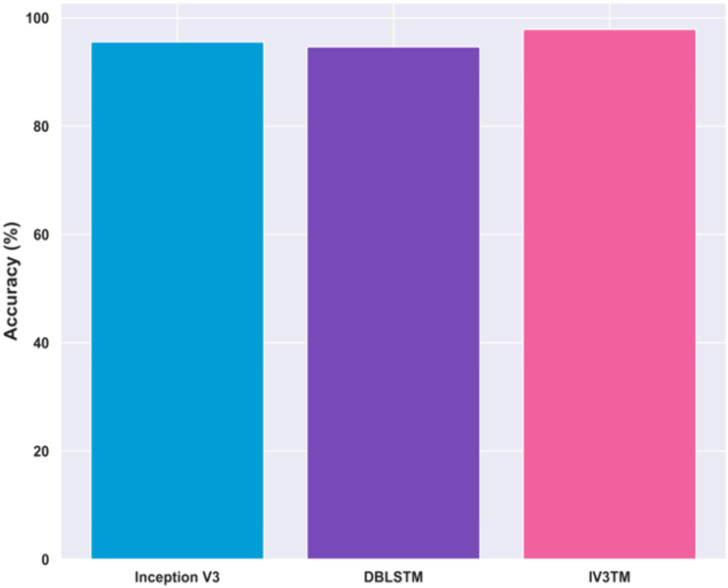
Ablation Study.

An ablation study is performed to evaluate the impact of each component in the proposed method on improving the brain tumor classification. In order to assess the contribution of each component, the performance of the proposed model is evaluated by removing certain components, and hence, the results are evaluated in terms of metric accuracy. From the ablation experiments, the proposed IV3TM model, combining the Inception V3 and DBLSTM, reports a high accuracy of 97.88%. Removing the DBLSTM from the proposed model reported a reduced accuracy of 95.56% for Inception V3 alone, indicating a performance drop of 2.36% over the proposed model. Further, the proposed model using the DBLSTM alone reports the accuracy of 94.64%, demonstrating the performance drop of 3.31% compared to the proposed model. Moreover, the proposed model, combining the Inception V3 and DBLSTM, effectively captures the complex patterns in the MRI data, resulting in high accuracy. Moreover, the ablation study shown in Fig 17 highlights the significance of the proposed IV3TM model in enhancing the brain tumor classification.

## 5. Conclusion

Brain tumors are one of the deadliest diseases with a high fatality rate. A significant difficulty in medical imaging of brain tumors can be found and identified thanks to aberrant cell proliferation, orientations, morphologies, and locations. To identify brain tumors, doctors frequently use the MRI imaging technique. A hybrid technique using brain MRI images was employed in this study using the Figshare brain tumor database and the Brain MRI image database to identify and classify the tumor. The created system uses hybrid Inception V3 and DBLTSM techniques to classify brain images as normal or cancerous tumors. The input photos were denoised and segmented utilizing the SqueezeNet segmentation strategy, and important characteristics were taken from the segmented images employing the Inception V3 model. The obtained features are put into a hybrid Inception V3 and DBTLSM models to classify MRI images of the brain. The outcome displays the most accurately classified Brain MR Images, and overall, this hybrid model strategy gave better outcomes. A brief inspection revealed that the proposed hybrid approach offers more efficient and enhanced categorization approaches.

### 5.1. Future scope

To extend this work, future research can explore the integration of attention mechanisms or transformer-based architectures to further enhance feature discrimination and model interpretability. Adopting end-to-end trainable unified networks that can jointly perform segmentation and classification by optimizing meta-heuristic algorithms may simplify the pipeline and reduce inference time. Additionally, incorporating multi-modal data could significantly enrich the feature space and improve diagnostic accuracy. Lastly, implementing Explainable Artificial Intelligence (XAI) tools will provide better decision-making, and the integration of Internet of Things (IoT) devices will enhance the real-time monitoring system, making it more acceptable in real-world clinical practice. Further, the research could also focus on improving the model’s robustness and adaptability through domain adaptation techniques in future research works.
